# Faculty of Nursing at Universidad de Antioquia: 70 years, a whole lifetime caring for lives

**DOI:** 10.17533/udea.iee.v38n3e01

**Published:** 2020-11-11

**Authors:** Lina María Vanegas Zuleta, Claudia Patricia Arrubla Lopera

**Affiliations:** 1 Nurse, Masters. Faculty of Nursing's Dean from 2016 to 2019. Email: lina.zuleta@udea.edu.co lina.zuleta@udea.edu.co; 2 Nurse, Masters. Faculty of Nursing's Dean from 2019 to 2022. Email: claudia.lopera@udea.edu.co claudia.lopera@udea.edu.co; 3 Universidad de Antioquia, Medellín (Colombia) Universidad de Antioquia Universidad de Antioquia Medellín Colombia

Seventy years have passed since Doctor Ignacio Vélez Escobar, in his possession speech as dean of the Faculty of Medicine at Universidad de Antioquia, on 23 August 1950, had the initiative of creating a new school of nursing. Thus, on 29 September of that year, Resolution Number 30 by the Directive Council at Universidad de Antioquia approved opening the School of Nursing, as dependence ascribed to the Faculty of Medicine. It operated under this figure until August 1975 when its creation resolution was repealed and it was determined that it would continue operating as a teaching unit at Universidad de Antioquia, but conserving its figure of School, which it kept until May 1981 when the same Council approved the transformation from School into Faculty.

For 2020, we had planned a great celebration for the 70^th^ anniversary of a Faculty of Nursing that has been pioneer in Antioquia, with national and international recognition, which offers three specializations, two master’s, and a doctorate; with important development in research and extension areas. For this celebration, we had additional conditions that supported us, among these that the Executive Council of the World Health Organization (WHO) declared 2020 as the International Year of the Nurse.(1) Further, the president of the International Council of Nurses highlighted that this decision by the WHO “will enhance the nurse’s figure and will highlight the need for well-prepared nurses, as well as the need to invest on recruitment and retention strategies; and will eliminate barriers to the development of the roles of advanced nursing that are demonstrating great efficacy in the expansion of universal health coverage".(2)

Moreover, the *Nursing Now*(3) campaign between 2018 and 2020 highlights that nurses are the heart of most of the health teams and that improving and promoting their role will improve health results of the citizens. All of the aforementioned within the framework of the celebration of 200 years since the birth of Florence Nightingale, considered the precursor of modern professional nursing and creator of the first conceptual model of the profession. However, within the framework of such well-deserved celebration, in March of the present year, the WHO declared that COVID-19 outbreak went from being an epidemic to a pandemic, a public health emergency of international importance with imminent consequences for health and the social and economic wellbeing of all the nations.

From that moment, without being something alien to our reason for being, protecting human lives became our maximum priority, the celebrations were reformulated, and the ways of teaching, doing research, and extension were reinvented. The new reality has led us to a transforming process where curricular flexibility gains importance; face-to-face methodologies have been displaced by virtuality and the students’ transit through their formation process is more dynamic, while activities and administrative actions that support teaching have become virtual.

We soon understood that our rhythm could not stop, but it was necessary to reformulate times, methods, and goals, while new commitments appeared to meet the current needs of our academic community and of our interest groups. Now, in honor of our slogan, **70 years, a whole lifetime caring for lives**, we are dedicated to displaying on the terrain why integral care, health promotion, prevention of complications, education for health, caring for the environment, interdisciplinary teamwork to maintain health and life are the center of interest of our formation and of our professional practice.

Thus, to meet the needs of our alumni, students, professors, administrative staff, and society in general, we opened our social networks to answer the principal questions made regarding the current pandemic situation; hence, the strategy **UdeA Nursing Caring for You, We care for each other at UdeA Nursing**. The answers are oriented according to up-to-date scientific information and guided by the Faculty’s academic groups.

Every week, the Faculty of Nursing, given the disciplined work by its academic groups, shares actions and recommendations of care and self-care for people and their families, through strategies, like personalized answers to questions guided via our social networks, infographics, educational videos and booklets. All this academic production can be consulted in our web page http://enfermeria.udea.edu.co. Additionally, in solidarity manner the theoretical-practical course is being given on nursing care to patients in critical state under COVID-19 protocol, directed at nursing professionals not specialized in critical care. Professors with expertise in distinct areas participate in 11 of the 16 work groups to analyze the social, labor, economic, territorial, and food safety implications due to COVID-19, a strategy generated from the University and led by the Vice-rectory of Research. The project: “Strengthening Mental Health in the Nursing Community” was begun and is led by a group of professors with training in family therapy and by the Welfare coordination in our dependence.

Today, 20 years after the start of this new century, also began the process of curricular modernization. Nursing professionals graduating from our faculty are critical in their practice, evidencing that the clinical and community care they provide is the fruit of the interaction of being, knowing, and knowing how to do; not as finished processes, but open to change and to the transformation’s characteristic of the uncertainty that the current reality imprints on their professional work. Now, we are shown a panorama where students do not dedicate their time solely to their professional formation, they are also workers. The professor, student and alumni are now citizens of the world. With academic-cultural internships, increasingly frequent. This, among other issues, has obligated the Faculty to think of a curricular transformation that contemplates homologations of the degree in other countries, analyze the number of credits in the study plan, denominate its courses, along with their thematic and practical contents according with the social, cultural, political, epidemiological dynamics that have marked big changes in daily life. The presence of chronic and reemerging diseases has obligated necessarily to a new dynamic and transforming formation process. As response to these new demands, a new proposal is introduced of curricular modernization, increasing the formation of nursing professionals from four to five years, seeking to continue on the leadership path at the national level and gaining global positioning.

We continue working for the future, with unwavering illusions for an autonomous professional exercise and with high qualification standards. The following are some of the principal aspects we have envisioned for our faculty:

Consolidation of a work group for issues of quality in graduate programs, for renovation of qualified registries and processes of quality accreditation. Participation in the curricular internationalization project led by the Vice-rectory for Teaching. Performance of feasibility studies to offer undergraduate and a specialization programs in a regional branch of the University. Elaboration of the master document of the specialization in Obstetric Nursing. Strengthening incoming and outgoing national mobility in light of the globalization of knowledge. The consolidation of environments that generate good living reactivates the work environment committee with different strategies that require and invite participation from all. We are advancing on the characterization of our alumni to offer accompaniment, especially during the initial years after graduation, which responds to their labor and formation needs.

In the line of managing the relationship with the environment, currently, we are undertaking some projects with the Mayor’s office of Medellín on very important themes for the city, like the elderly, disability, care in emergency services, and health promotion and disease prevention with differential approach. We are managing a project to accompany and update the guidelines of Primary Health Care for the department of Antioquia. We are undertaking strategies of pedagogical development that support the implementation process of curricular transformation. The current conditions that lead to diminishing the professor/student ratio and the time of permanence in the practice sites require greater and better equipping of our skills laboratory, thus, we advance in the project of renovation of simulators for academic enhancement in the undergraduate, graduate, and extension programs.

We are working on improving the conditions of physical infrastructure of the Faculty and on starting the construction of the new building in the lot next to our facilities; currently, its designs are in approval phase. Research and reflection, as an activity characteristic of universities, tends to the search, production, dissemination and appropriation of knowledge and of its logics. This is how the *Cuaderno de la Facultad* (Faculty Notebook) emerges, which will be a digital medium of dissemination of the academic production by students and professors; a component to make visible the research of our action plan, under the leadership by the head of the Research Center and with participation from a group of five professors and one student. The *Cuaderno* is framed by the guidelines that in research are given from the University and the Faculty on the importance of disseminating knowledge for disciplinary development and on the urgent formation necessity of students in research, critical reading and writing, further revealed in the diagnostic phase of the recent process of curricular transformation.

So many projects carried out and so many still pending account for the commitment we have with undergraduate and graduate formation, as well as with the generation of human knowledge, which has always been the motor of social development that seeks to satisfy the desire to learn as an inherent condition of the very life of each subject. This search for knowledge is transferred among intergenerational relations to consolidate learning and an episteme that is affirmed at every moment of the institutional life of our Faculty of Nursing.

Although we are aware about having had to adapt to this new reality, we wish to dream with a fraternal face-to-face meeting. For this reason, as of 29 September, day to commemorate the official creation of our esteemed Faculty, we will continue celebrating until reaching the 71^st^ anniversary, hoping that the evolution of the COVID-19 pandemic allows us the opportunity to meet in 2021 to celebrate life.

The history of these 70 years is the construction of the work by students, graduates, retirees, professors, employees, and allies, all are leading players.

Thank you, Faculty of Nursing for 70 years! For a whole lifetime caring for lives!
